# Hypogonadotropic Hypogonadism Associated with Hereditary Hemorrhagic Telengiectasia

**DOI:** 10.1155/2013/465376

**Published:** 2013-04-04

**Authors:** Scarano Valentina, De Santis Daniele, Suppressa Patrizia, Lastella Patrizia, Lenato Gennaro Mariano, Triggiani Vincenzo, Sabbà Carlo

**Affiliations:** ^1^Neurology Unit, “S. Maria del Pozzo” Hospital, Somma Vesuviana, 80049 Naples, Italy; ^2^Center for Rare Diseases, “Clinica Medica Frugoni”, University Hospital of Bari, 70124 Bari, Italy; ^3^Unit of Endocrinology, University Hospital of Bari, 70124 Bari, Italy

## Abstract

A 65-year-old man was referred to our clinic for the rehabilitation of right hemiparesis caused by ischaemic stroke. Hypertension, postphlebitic syndrome of lower limbs, frequent nose bleeding, and anemia were present in his history; in his adolescence, he was treated for idiopathic hypogonadotropic hypogonadism. Further investigations have revealed also microsomia, suggesting a clinical diagnosis of Kallmann syndrome, that is, an association, possible in males and females, of hypogonadotropic hypogonadism with olfactory deficits. A definite diagnosis of hereditary hemorrhagic telangiectasia was made based on clinical criteria and confirmed by genetic analysis.

## 1. Introduction

Hereditary hemorrhagic telangiectasia (HHT) is an autosomal dominant disorder of angiodysplasia, affecting 1 in 5–8,000 people, most commonly caused by mutations in *ENG* or *ALK1/ACVRL1* gene [1]. A definite clinical diagnosis of HHT is made in the presence of at least three separate manifestations: spontaneous recurrent nosebleeds, mucocutaneous telangiectases (multiple at characteristic sites: finger pulps, lips, oral mucosa, and tongue), visceral involvement (gastrointestinal, pulmonary, hepatic, cerebral, or spinal arteriovenous malformations, AVMs), and family history (an affected first-degree relative) [2]. Spontaneous recurrent nosebleeds are the most common and usually earliest clinical feature, even though they may also arise later in age. Telangiectases of the skin and mucosae affect up to 90% of patients by the fourth decade of life. Visceral AVMs in pulmonary, hepatic, or cerebral circulation are found at a variable rate, depending upon mutated gene and employed screening technique [3]. Complications of HHT include anemia from chronic gastrointestinal and nasal haemorrhaging, brain abscess/stroke, liver disease, severe dyspnoea, and pulmonary haemorrhage. 

Male idiopathic hypogonadotropic hypogonadism (MIHH) has a prevalence of 1 case per 4–10,000 individuals [4]; 60% of patients with hypogonadotropic hypogonadism present anosmia, such association being known as Kallmann syndrome (KS). MIHH is characterized by partial or complete lack of pubertal development, due to, defect in synthesis, secretion, or action of gonadotropin-releasing hormone (GnRH). Laboratory investigations reveal low or inappropriate normal values of circulating LH and FSH, despite normal secretion of other pituitary hormones and unremarkable MRI findings of hypothalamic-hypophyseal tract and olfactory bulbs. Defects in olfactory and GnRH-releasing neuron migration account for KS clinical features. Many gene mutations have been described in association with KS and other forms of hypogonadotropic hypogonadism [4]. To our knowledge, the case here described is the first one presenting MIHH in association with HHT, clinically defined and genetically confirmed.

## 2. Case Report

**Figure 1:**
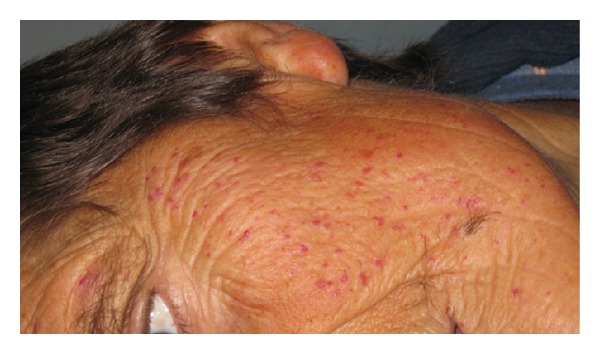
Telangiectases on face.

**Figure 2:**
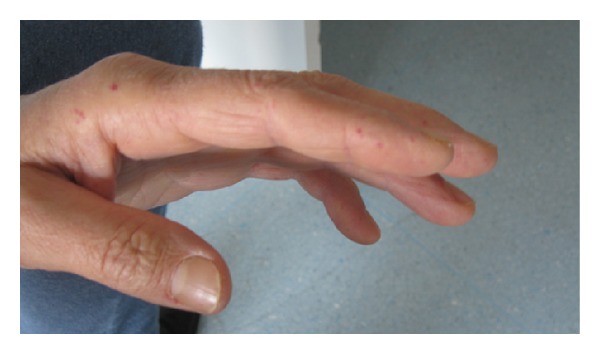
Telangiectases on fingers.

**Figure 3:**
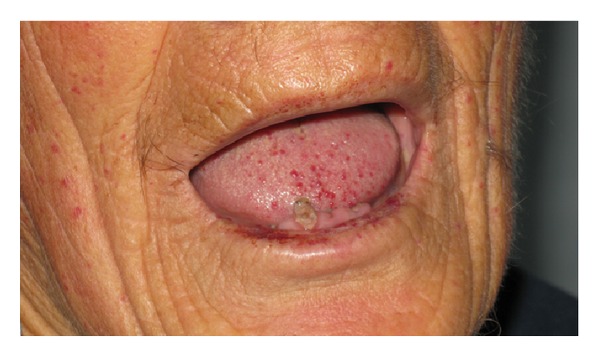
Telangiectases on tongue.

A 65-year-old man was admitted to our clinic to undergo neurorehabilitation. He presented right hemiparesis as a result of a lacunar stroke which had occurred 20 days before. His past medical history was positive for hypertension, postphlebitic syndrome of lower limbs, previous TIA, frequent nose-bleedings from infancy, and hypogonadotropic hypogonadism. He felt tired but he was not worried about absence of libido and sexual activity. At medical examination, he presented tall stature, eunuchoid habitus, gynecoid fat deposition, and scarce body hair. Testes were small and soft at clinical examination. Numerous telangiectases were present on the face (Figure 1), the fingers (Figure 2), and the tongue (Figure 3). 

**Figure 4:**
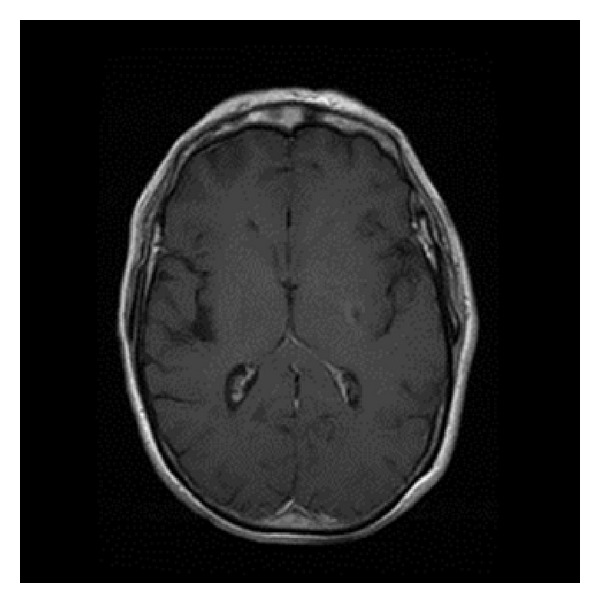
T1-weighted brain MRI showing ischaemic lesion of left posterior putamen.

**Figure 5:**
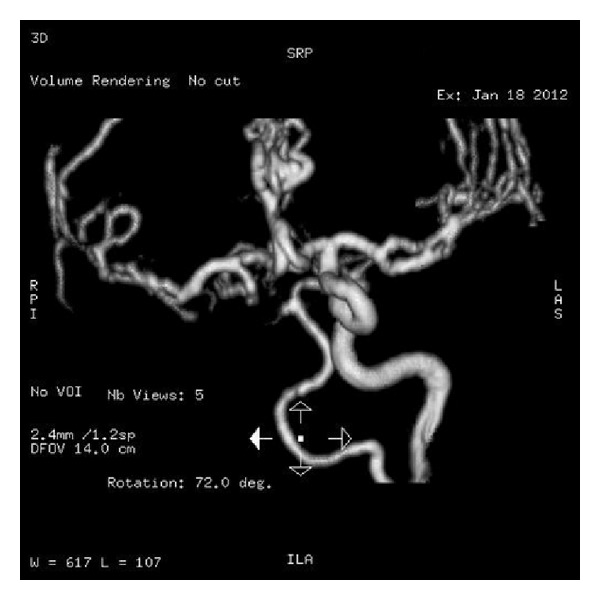
Brain Angio MRI showing occlusion of left internal carotid.

**Figure 6:**
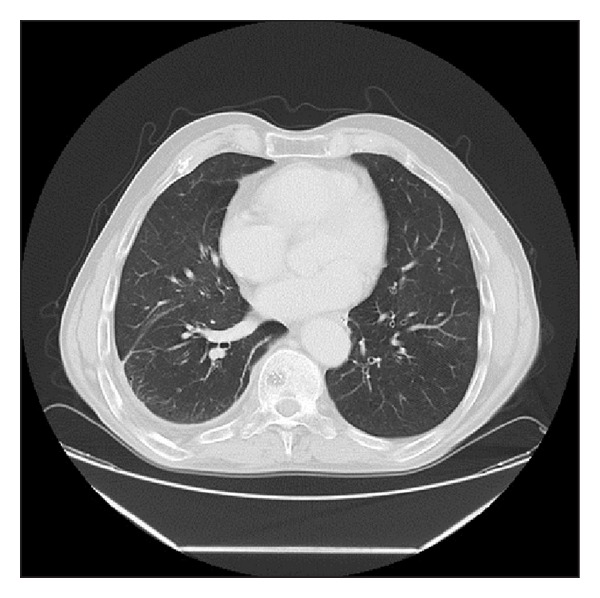
Chest CT revealing a large pulmonary AVM in the basal segmento flowe right lobe.

Hormonal screening was consistent with hypogonadotropic hypogonadism: FSH 2.29 (1.4–18.1) mU/mL and LH 0.4 mU/mL (1.5–9.3), testosterone 25.4 ng/dL (241–827), sex hormone binding globulin 48.7 nmol/L (9.0–55.0), prolactin 3.91 ng/mL (2.1–17.7), estradiol 11.8 pg/mL (0–40), DHEA-S 52.10 ug/dL (40.8–405.4), and progesterone 0.35 ng/mL (0.28–1.22); his hormonal profile seemed to exclude pituitary deficiency other than gonadotropin abnormalities: ACTH 44.7 pg/mL (5.0–77.0), plasma cortisol 16.6 ug/dL (9.0–23), insulin 9.6 microU/mL (3.2–16.3), C-peptide (2.34 ng/mL (0.8–4.2), GH 0.5 ng/mL (0.1–8.0), and IGF-1 135.0 ng/mL (54.0–499.0). Prostatic hypotrophia and osteopenia were found by prostatic US and DEXA evaluation, respectively; karyotype was normal (46, XY). Olfactory function fell in the range of microsomia according to UPSIT (University of Pennsylvania Smell Identification Test). Brain MRI showed ischemic lesion of left posterior putamen (Figure 4) and occlusion of left internal carotid (Figure 5), with no abnormalities detectable in the hypothalamic-hypophyseal region. Chest CT revealed a large pulmonary AVM in the basal segment of lower right lobe (Figure 6) (feeding artery of 8 mm in diameter). Vascular shunts were also detected in the liver by abdominal US. Full molecular analysis of both HHT-causing genes permitted the identification of a missense mutation in exon 8 of *ENG* gene (c.1088G>C, p.Cys363Ser), which has never been reported previously. The patient refused to be submitted to testosterone replacement therapy (TRT).

## 3. Discussion 

The patient described here matches three out of four clinical criteria for a definite diagnosis of HHT, according to Curacao criteria published in 2000 [2]. Frequent epistaxes have been reported in his history from infancy. Mucocutaneous telangiectases were evident at clinical examination whereas visceral involvement (lung and liver AVMs) was revealed by imaging investigation. A diagnosis of HHT had not been suspected, until his lung AVM became suddenly symptomatic with a brain stroke likely due to paradoxical embolization, in the absence of any respiratory manifestation, as often reported [5]. His hepatic AVMs are clinically silent, which is typical of HHT-related liver involvement [1, 3]. Molecular screening disclosed an *ENG* missense mutation, probably arising as a *de novo* mutational event in either parent, given the absence of HHT-related signs in his ascendants and numerous siblings. The *ENG* c.1088G>C mutation, predicting a change of Cys-363 into a serine residue, has never been reported before and is not listed within the HHT mutation database [6]. However, a mutation affecting a different nucleotide within the same codon has been previously published (c.1087T>A), which determines the same amino acidic change in the predicted polypeptide (p.Cys363Ser). Such infrequent scenario is explained by the peculiarity of the serine-coding triplets, which consist in two disjunct nonadjacent codon clusters. The finding that distinct adjacent mutational events, leading to the same amino acid change, are responsible for HHT actually points out to a key role played by Cys-363 in endoglin expression/function, as supported by both structural [7] and functional studies [8]. 

In males, the prevalence of hypogonadotropic hypogonadism is thought to be from 1/4,000 to 1/10,000 [4], half of which being accounted for by syndromic forms (KS or less frequent disorders). The features of hypogonadotropic hypogonadism in our patient were strongly suggestive for KS, in the light of the olfactory deficit detected by appropriate test [4]. Other disorders possibly underlying hypogonadotropic hypogonadism in this patient were considered unlikely. Multiple pituitary deficiency or hyperprolactinemia could be ruled out due to the previously reported hormonal profile. Furthermore, there were no MRI abnormalities affecting hypothalamic-pituitary region. Finally, Klinefelter's syndrome, the commonest form of male adult hypogonadism, was excluded by karyotype analysis, testicular phenotype, and his hormonal profile, as Klinefelter's syndrome is associated to increased, rather than decreased, gonadotropin levels [9]. TRT aiming at restoring normal serum testosterone levels and improving clinical conditions was proposed to the patient. In fact, there is evidence in hypogonadic patients about the positive effects of TRT on muscle mass and strength, bone density, fat distribution, metabolic parameters, cardiovascular risk, and development and maintenance of male sexual features, erection, and libido [10]. Furthermore, testosterone preparations leading to stable normal testosterone serum levels have become available in the last decade. The patient, however, after being adequately informed about the possible advantages of TRT, refused the treatment. 

Both HHT and MIHH are rare genetic disorders, with a similar prevalence, ranging from 1 : 4–5,000 to 1 : 10,000 [1, 4], the latter affecting only males. Our patient was affected by both of these conditions, likely due to a coincidence of two infrequent events. Assuming that the two disorders are not causally related, such a coincidence is expected to occur with a frequency from 1 : 20,000,000 to 1 : 100,000,000 in the male general population. Since the Italian population is represented by ~30,000,000 male individuals, only 1-2 subjects are expected to harbor this combination of rare diseases. While HHT was presumably determined by a *de novo* mutational event in the *ENG* gene, the cause underlying MIHH may consist in recessive mutation(s) segregating in the patient's family, since a number of mutations have been reported in several autosomal or X-linked genes [4]. 

## References

[1] Faughnan ME, Palda VA, Garcia-Tsao G (2011). International guidelines for the diagnosis and management of hereditary haemorrhagic telangiectasia. *Journal of Medical Genetics*.

[2] Shovlin CL, Guttmacher AE, Buscarini E (2000). Diagnostic criteria for hereditary hemorrhagic telangiectasia (Rendu-Osler-Weber syndrome). *American Journal of Medical Genetics*.

[3] Sabbà C, Pasculli G, Lenato GM (2007). Hereditary hemorrhagic telangiectasia: clinical features in ENG and ALK1 mutation carriers. *Journal of Thrombosis and Haemostasis*.

[4] Young J (2012). Approach to the male patient with congenital hypogonadotropic hypogonadism. *The Journal of Clinical Endocrinology and Metabolism*.

[5] Pierucci P, Lenato GM, Suppressa P (2012). A long diagnostic delay in patients with Haereditary Hemorrhagic Telangiectasia: a questionnaire-based retrospective study. *Orphanet Journal of Rare Diseases*.

[6] HHT Mutation Database http://arup.utah.edu/database/hht/index.php.

[7] Paquet ME, Pece-Barbara N, Vera S (2001). Analysis of several endoglin mutants reveals no endogenous mature or secreted protein capable of interfering with normal endoglin function. *Human Molecular Genetics*.

[8] Llorca O, Trujillo A, Blanco FJ, Bernabeu C (2007). Structural model of human endoglin, a transmembrane receptor responsible for hereditary hemorrhagic telangiectasia. *Journal of Molecular Biology*.

[9] Rey RA, Gottlieb S, Pasqualini T (2011). Are Klinefelter boys hypogonadal?. *Acta Paediatrica*.

[10] Giagulli VA, Triggiani V, Corona G (2011). Evidence-based medicine update on testosterone replacement therapy (TRT) in male hypogonadism: focus on new formulations. *Current Pharmaceutical Design*.

